# Seroprevalence of rubella virus antibodies among pregnant women in the Center and South-West regions of Cameroon

**DOI:** 10.1371/journal.pone.0225594

**Published:** 2019-11-21

**Authors:** Nadesh Ashukem Taku, Valantine Ngum Ndze, Emily Abernathy, LiJuan Hao, Diane Waku-Kouomou, Joseph P. Icenogle, Samuel Wanji, Jane-Francis K. T. Akoachere

**Affiliations:** 1 Department of Microbiology and Parasitology, Faculty of Science, University of Buea, Buea, Cameroon; 2 Faculty of Medicine and Biomedical Sciences, University of Yaoundé I, Yaoundé, Cameroon; 3 Centers for Disease Control and Prevention, Atlanta, Georgia, United States of America; 4 IHRC Inc, Atlanta, Georgia, United States of America; South African Medical Research Council, SOUTH AFRICA

## Abstract

Rubella infection in early pregnancy can lead to miscarriages, fetal death, or birth of an infant with congenital rubella syndrome (CRS). In Cameroon, like in many developing countries, rubella surveillance is not well-established. The aim of this study was to determine the prevalence of rubella virus specific antibodies among pregnant Cameroonians. We conducted a cross-sectional study for rubella infection among pregnant women attending antenatal clinics in the Center and South-West regions of Cameroon. Demographic data and blood were collected and tested for rubella specific antibodies (IgG and IgM), and for the IgM positive cases, IgG avidity and real time PCR was done. From December 2015 to July 2017, 522 serum samples were collected and tested from pregnant women. The seroprevalence of rubella specific IgG was 94.4%, presumably due to immunity induced by wild-type rubella virus. The seroprevalence of rubella specific IgM was 5.0%, possibly indicating rubella infection. However, IgG avidity testing of the IgM positive cases detected high avidity IgGs, ranging from 52.37% to 87.70%, indicating past rubella infection. 5.6% (29/522) of the participants had negative results for IgG to rubella virus, indicating susceptibility to rubella infection. None of the participants had received a rubella containing vaccine (RCV), but 51% (266/522) of the pregnant women lived in a house with a child with records of at least one dose of RCV. Rubella virus RNA was not detected in the urine of any IgM positive case. Findings from this study show that rubella infection is significant in Cameroon. Some pregnant women are still susceptible to rubella infection. For a better management of rubella infection in pregnancy in Cameroon, consideration should be taken to investigate for IgG-avidity test in cases with positive rubella IgM result to distinguish between recent from past rubella infection.

## Introduction

Rubella virus infection is transmitted by respiratory droplets and causes a generally mild disease characterized by a rash and fever, primarily in children [1]. Although the disease affects both males and females, it is a disease of public health importance in pregnant women causing major problems such as spontaneous abortions, miscarriages, stillbirths, and congenital defects including hearing impairment, heart defects, cataracts known as congenital rubella syndrome (CRS) [2]. From just before conception and during the first 8–10 weeks of gestation, rubella infection may cause multiple fetal defects in up to 90% of cases [3].

Rubella virus, the sole member of the *Rubivirus* genus in the *Togaviridae* family, is a positive-polarity ribonucleic acid virus. Both vaccination and natural infection result in life long immunity. This vaccine-preventable disease is among the small number of viral diseases considered to be potentially eradicable [4, 5]. The primary objective of rubella-control programs is prevention of congenital rubella virus infection, which can result in CRS. Cameroon introduced rubella containing vaccine (RCV) into the Expanded Program on Immunization (EPI) in 2015 through a national measles and rubella mass vaccination campaign for children under 15 years [6]

Previous reports about rubella in Cameroon indicated a prevalence of rubella IgG antibodies of 83.9% in women of reproductive age in 1992 [7] and 88.8% in women obtaining antenatal care in 2008 [8]. Recently, a study in the West region reported a prevalence of rubella IgG antibodies of 93.4% among pregnant women [9]. Additionally, a report indicated a prevalence of 1.3% of rubella IgM antibodies in febrile infants in Cameroon [10]. Prior to rubella vaccine introduction by the EPI, rubella IgM seroprevalence was estimated at 9.3% in measles negative sera [11]. There are two reports on CRS in Cameroon, a case report [12] and a study of CRS in school children [13]. Unfortunately, there is no recent report on rubella in pregnant women in the Center region. In addition, there is no report on rubella IgM seroprevalence and IgG avidity in pregnant women in Cameroon. Therefore, the goal of the current study was to re-evaluate rubella infection in pregnant women in Cameroon and to distinguish recent from past rubella infection using rubella IgG avidity testing.

## Material and method

### Study design, sites and duration

A cross-sectional study was conducted in the Center and South-West regions, to determine the seroprevalence of rubella IgG antibodies, rubella infection (IgM antibodies), and to distinguish recent infection from past infection by IgG avidity. Pregnant women were recruited from hospitals in the Center region (Yaoundé Gyneco-Obstetric and Pediatric hospital, the Mother and Child Center of the Chantal Biya Foundation and the Quality Healthcare unit) and South-West region (Buea Regional Hospital, Mount Mary Hospital and 5 health centers in the Buea health district). The recruitment was conducted from December 2015 to July 2017.

### Study participants

The study participants were pregnant women who visited the respective hospitals’ antenatal care (ANC) clinics during the study period. All participants gave informed consent and the required amount of blood and urine for laboratory analysis.

### Sampling and sample size

Convenience sampling was used to recruit participants. The sample size was calculated using the Lorenz formula by considering a 95% confidence interval, cut-off value at 1.96, 0.025 margin of error and a proportion of 9.3% from a previous rubella study in Cameroon [11]. After calculations, at least 518 participants were supposed to be enrolled for the study.

### Data collection

Data were collected using a questionnaire that included participant information, clinical characteristics, family history and laboratory findings. Five milliliters of blood were collected from the subjects by venipuncture into labelled sterile tubes and allowed to clot undisturbed at room temperature. Sera was separated by centrifugation at 3,000 revolutions per minute (rpm) for five minutes and stored in two aliquots at -20°C. Urine samples were also collected in sterile urine cups and stored in two aliquots at -20°C. Samples were shipped on dry ice to the Viral Vaccine Preventable Disease Branch of the Centers for Disease Control and Prevention (CDC; Atlanta, Georgia) and stored at -70^o^ C until laboratory analysis.

### Laboratory testing

#### IgG and IgM enzyme-linked immunosorbent assay (ELISA) for rubella virus -specific antibody

Rubella virus specific IgG antibody detection was performed on serum samples using the Zeus ELISA rubella IgG kit (Zeus Scientific, New Jersey, USA) per manufacturer’s instructions. Rubella virus-specific antibody IgM detection was performed using the Diamedix immunosimplicity^®^ Is-Rubella IgM capture test kits (Diamedix Corporation, Florida, USA) per manufacturer’s instructions.

#### Measurement of rubella virus-specific IgG antibody avidity

For the IgM positive serum, rubella IgG antibody avidity testing was performed using the Zeus ELISA rubella IgG kit (Zeus Scientific, New Jersey, USA). The measurement of rubella IgG antibody avidity was used to differentiate between recent and remote rubella infection. Avidity differences were detected by using 35mM diethylamine (DEA), a protein binding inhibitor in the washing step of the indirect ELISA for rubella IgG. DEA releases antibodies with low avidity. An avidity index was calculated by comparing the optical density obtained with and without a DEA wash [14]. The cut-off between low and high avidities was established by the use of standardized sera and the Zeus ELISA rubella IgG kit. Serum samples were tested in duplicate.

#### RNA extraction and diagnostic real time polymerase chain reaction (PCR)

Viral RNA was extracted from the urine of IgM positive persons using a Viral RNA Mini kit (Qiagen, Valencia, CA), per the manufacturer's instructions. Extracted viral RNA was stored at -70°C until the diagnostic real time RT-PCR was performed.

A real-time (TaqMan^®^) RT-PCR assay was used for the detection of rubella virus RNA using primers and probe specific to a region in the non-structural protein (NSP) coding region using the ABI 7500 Real-Time Thermocycler (Applied Biosystems, USA). Real time PCR was performed using Superscript III One-Step qRT-PCR (Invitrogen, Carlsbad, CA), to amplify a 154-nucleotide region) using a Rubella virus forward primer (RV98F [5′-GGC AGT TGG GTA AGA GAC CA-3′]) and a reverse primer (RV251R [5′-CGT GGA GTG CTG GGT GAT-3′]). RNA samples were also tested using RNase P forward primer (HURNASE-P-F): 5’-AGA TTT GGA CCT GCG AGC G-3’ and a reverse primer (HURNASE-P-R): 5’-GAG CGG CTG TCT CCA CAA GT-3’ [15]. Briefly, after the reverse transcription step for 30 min at 55°C and denaturation of cDNA for 5 min at 95°C, the reaction mixtures were incubated for 35 cycles at 95°C for 30s, 60°C for 30s, and final extension at 72°C for 1 min, followed by 72°C for 5 min.

### Data analyses

Data were entered and statistical analysis was performed using Microsoft Excel 2016 and Epi Info version 3.5. Data were cleaned and descriptive analysis was done. Bivariate analyses were done using χ2 or Fisher’s exact tests for categorical variables to find key determinants of rubella infection in Cameroon. A 95% Confidence Interval (CI) was calculated for the Odd ratio (OR), and values of p<0.05 were considered statistically significant. Logistic regression analysis was used to estimate OR of IgG and IgM seropositivity as outcome variable with age, sex, setting, pregnancy, region, presence of child who has taken RCV, number of children living in same house and tribe as explanatory variable.

### Ethical issues

The study protocol was approved by Cameroon National Ethics Committee N0 2017/11/957/CE/CNERSH/SP. It also received approval from other institutional review boards such as the Institutional Review Board of the Cameroon Baptist Convention Health Board, the Yaoundé Gyneco-Obstetric and Pediatric Hospital, the Centre Hospitalier Essos and the St Elizabeth General Catholic Hospital Shisong. Research authorizations were obtained from each hospital and health districts where participants were recruited. Consent was also obtained from parents or guardians of the minors included in the study. Informed consent was obtained from all study participants.

## Results

### Demographic characteristics of participants

Five hundred and twenty-two (522) pregnant women (Table 1) were enrolled from the Center [39.5% (206/522)] and South-West [60.5% (316/522)] regions of Cameroon. The mean (±SD) age of 26.6 ± 5.2 years was observed. Most of the pregnant women (37.9%; 198/522) were in the 24–28 years age group. Some of the women, 16.7% (87/522) were in the first trimester of pregnancy. Among the pregnant women, 36.0% (188/522) lived in a house with no child during their pregnancy. 51.0% (266/522) of the pregnant women lived in a house in which at least one child had received a rubella containing vaccine. None of the pregnant women had received an RCV.

**Table 1:** Association between rubella antibodies and characteristics of pregnant participants.

Characteristics	No. tested (N = 522)	IgG positive (N = 493)			
	n(%)	n(%)	COR	AOR	p-value
**Regions**					
Center	206(39.5)	199(40.4)	2,12(0,89–5,07)	0.21(0.062–0.736)	0.015
South-West	316(60.5)	294(59.6)	1		
**Age group (years)**					
14–18	22(4.2)	22(4.5)	-		0.18
19–23	136(26.1)	126(25.6)	1		
24–28	198(37.9)	188(38.1)	1,49(0,60–3,68)		0.38
29–33	115(22.0)	110(22.3)	1,74(0,57–5,26)		0.31
≥ 34	51(9.8)	47(9.5)	0,93(0,27–3,11)		0.90
**Age of pregnancy**					
First trimester	87(16.7)	85(17.2)	1		
Second trimester	311(59.6)	295(59.8)	0,43(0,09–1,92)		0.26
Third trimester	124(23.8)	113(22.9)	0,24(0,05–1,11)		0.09
**Number of children living in the same house**					
0	188(36.0)	176(35.7)	1		
≥1	334(64.0)	317(64.3)	1.27(0.59–2.72)		0.67
**Any child in house with RCV**					
Yes	266(51.0)	255(51.7)	1,51(0,66–3,45)	3.58(1.17–11.004)	0.025
No	212(40.6)	199(40.4)	1		
Unknown	44(8.4)	39(7.9)	0,5(0,17–1,51)		0.36

**Key:** COR: Crude Odds Ratio; CI: Confidence Interval; AOR: Adjusted Odds Ratio; N: Number; n: subtotal; %: proportion of n/N

### Rubella clinical signs and seroprevalence of rubella virus antibodies

#### Prevalence of rubella IgG and IgM antibodies

**Fig 1:**
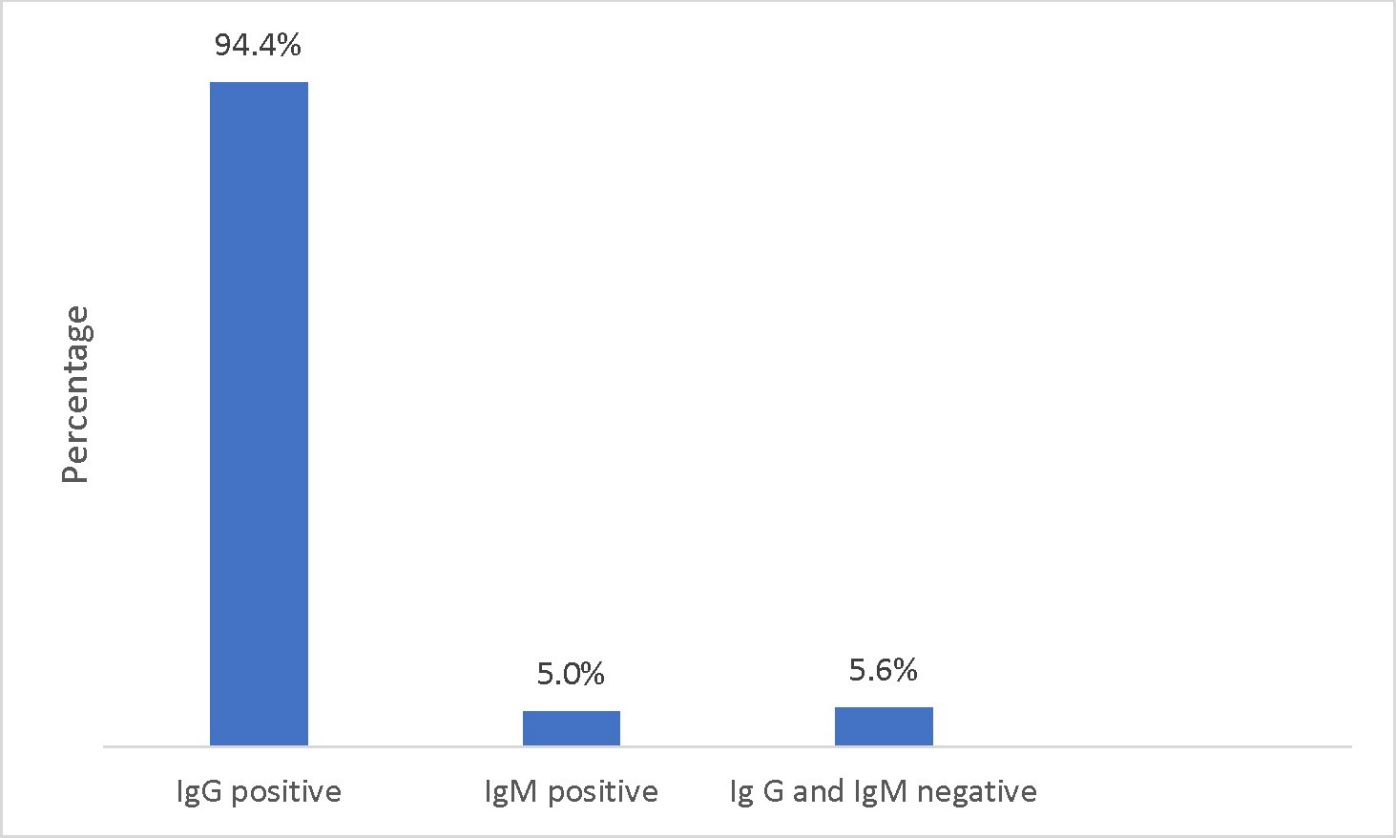
Rubella immune status of pregnant women in the center and South-West regions of Cameroon. IgG positive: indicates the proportion of participants with indication of protective immunity; IgM positive indicates possible rubella infection; IgG and IgM negative indicates no immunity to rubella.

The overall seroprevalence of rubella specific IgG antibodies at the time of data collection was 94.4% (493/522). The overall seroprevalence of rubella specific IgM was 5.0% (26/522). According to the present study, 5.5% (29/522) of the total participants had no indication of immunity to rubella virus and tested negative for both IgG and IgM antibodies (Fig 1).

#### Association between rubella antibodies and characteristics of pregnant participants

Among the pregnant participants, 94.4% (493/522) were IgG positive with the South-West region having the higher prevalence of 59.6% (294/493). 5.0% (26/522) of the pregnant participants were IgM positive within the Center region having the higher prevalence of 65.4% (17/26) and the South-west the lower with 34.6% (9/26). 5.6% (29/522) of the pregnant participants were IgG and IgM negative. Pregnant participants from the Center region were two times more likely to have a positive IgG (COR: 2.12, 95% CI: 0.89–5.07, p = 0.015). The relationship between rubella IgG and the other sociodemographic characteristics (age group, age of pregnancy, number of children living in the same house, any child with RCV and tribe) did not show any statistically significant association. However, rubella IgG was statistically associated to having any child with RCV in the same house during pregnancy (COR: 3.58, 95% CI: 1.17–11.004, p = 0.025).

#### Rubella IgG avidity

In this study, all the IgM positive cases had past rubella infection. Of 26 serum samples tested for rubella IgG avidity, all samples had high avidity rubella IgG antibodies which varied from 52.37% to 87.70% with an average of 69.87% (± 8.19%).

#### Prevalence of rubella IgG and IgM antibodies among pregnant women presenting with rubella clinical signs

In this study, 19% (99/522) of the pregnant participants presented with rubella clinical signs. 7.5% (37/493) of the IgG positive pregnant women had fever, 4.1% (20/493) had rash, 7.5% (37/493) had conjunctivitis and 0.2% (1/493) had lymphadenopathy. There was no statistically significant association between rubella IgG positivity and the presence or absence of any clinical presentation. Among the pregnant women presenting with rubella clinical signs, 7.7% (2/26) of the IgM positive participants presented with fever; 7.7% (2/26) presented with rash; 11.5% (3/26) presented with conjunctivitis and no participant positive to rubella IgM presented with lymphadenopathy. However, the number of IgM positive cases was too few for any significant statistical analyses between IgM positivity and rubella clinical signs.

### Detection of rubella virus by real time PCR

RNA was extracted from a total of 14 urine samples from the pregnant participants with positive IgM results. No rubella virus was detected from any of these samples using real time PCR.

## Discussion

This study investigated the seroprevalence of rubella among Cameroonian pregnant women attending ANC in the Center and South-West regions. Rubella antibodies (IgG and IgM) were detected using ELISA and rubella IgG avidity testing was done to determine the status of rubella infection. The findings showed that 94.4% of the pregnant women were positive to rubella IgG. This confirms previous exposure to wild type rubella virus [1, 16] since, none of the participants in this study had ever received any rubella containing vaccine. The seroprevalence of rubella IgG among pregnant women in this study (94.4%) is higher than that of previous studies in Cameroon where rubella seroprevalence varied from 83.9% to 93.4% among pregnant women attending ANC in hospitals [7, 9]. Other studies in Cameroon show recent rubella virus transmission and reported that persons aged ≥15 years were also more likely to have rubella infection than children under one [11]. Since none of the participants had received any rubella containing vaccine, our results suggest that most of the participants were probably exposed to the virus through natural rubella infection. This high level of rubella seroprevalence in Cameroon likely reflects significant transmission of rubella virus in the country. This is similar to the situation reported in other African countries such as Burkina Faso [17], where a prevalence of 95% was found; Sudan [18] with a prevalence of 95.1%; Nigeria [19] where a prevalence of 91.54% was found and in Tanzania [20], where a prevalence of 90.4% was reported.

Furthermore, as observed in other studies, all the other socio-demographic characteristics were not associated to rubella seroprevalence [16, 17, 21], except for living in the same house with a child with RCV during pregnancy. Even though there is lack of evidence of contact spread from vaccinated individuals [22], living in the same house with a vaccinated infant was protective against rubella infection during pregnancy [16]. However, more information on the timing of rubella vaccination of the children would be needed as the vaccine virus would only be shed for a short time (such as 1 month) after the vaccination was received.

This study shows a lack of immunity against rubella among a small proportion of pregnant women in Cameroon as the proportion of susceptible individuals was 5.6%. Despite the high prevalence of rubella IgG in Cameroon, some pregnant women are still at risk of rubella infection during pregnancy which might lead to the birth of infants with CRS. Presently, some young women of child bearing age are still unprotected from rubella which is consistent with some observations of CRS in Cameroon in the past few years [13]. The main aim of rubella vaccination is to prevent CRS. A measles and rubella containing vaccine was administered for the first time in Cameroon in 2015 to children between 9 months and 14 years during a mass vaccination campaign [6]. We recommend that this strategy employed by the EPI should be re-enforced by also vaccinating young women of child bearing age.

Clinical diagnosis of rubella is unreliable [23], making laboratory diagnosis an important component in the diagnosis of primary rubella infection during pregnancy. In this study, there was no significant association between the presence or absence of clinical signs and rubella IgG positivity. In Ethiopia, pregnant women without maculopapular rash had 2.5 times protective IgG antibody than those who had maculopapular rash [16].

It is recommended to test pregnant women for IgM only if symptoms are evident, if contact with a known case occurred, or if an outbreak is occurring in the region but no pregnant woman should have rubella diagnosed on the basis of a single positive rubella specific IgM test result[24]. Occasionally, rubella IgM may persist for months or years after infection [25]. Additionally, a small percentage of false positive IgM results are seen with all ELISA IgM kits and cross reactivity has been seen with rheumatoid factor and other diseases such as parvovirus B19 [25]. The positive predictive value of IgM tests declines as rubella incidence declines [25] so the need for supplementary assays to accurately diagnose rubella will become more common as Cameroon progresses toward rubella control. This study is the first study to document seroprevalence of rubella IgM in pregnant women in Cameroon. The prevalence of rubella IgM was 5.0% among the pregnant women. This falls within the range of acute rubella infection in Africa observed to vary from 0.3% in pregnant women in Mwanza, Tanzania; to 45.1% in children (1–10 years) in Jos, Nigeria [20].

In cases where a possible false positive rubella IgM result is suspected, one supplemental assay that can be used to distinguish primary infection from remote infection is the measurement of IgG avidity [14]. We recommend that the management of pregnant women presenting with a positive rubella specific IgM result should be followed up. The timing of serum collection is important for pregnant women. If the serum is collected in the first trimester and the avidity is high, rubella infection likely occurred prior to the start of the pregnancy so danger to the fetus is very low. However, if the serum was collected in the second or third trimester, the avidity could already be high if a rubella infection occurred in the first trimester so danger to the fetus cannot be ruled out by high avidity. The interpretation of the results in this study is that past rubella infection had occurred in all IgM positive individuals, as the avidity results were above 50%. Even though all the avidity results were high, it does not completely exclude the possibility of a recent secondary infection. Secondary infections cannot be confirmed without two serum samples with one collected close to onset and a second sample collected one to two weeks later, which was not done in this study. The risk of defects in the fetus due to secondary infection is substantially less than the risk from primary rubella [26]. For IgM positive women presenting with symptoms consistent with rubella, another option for laboratory confirmation of disease is to use ribonucleic acid (RNA) detection by reverse-transcription polymerase chain reaction (PCR) assays on samples such as throat swabs or urine. In this study, 14 urine samples from the IgM positive women were tested with negative results. Real time PCR is most likely to be positive for samples collected within 4 days of disease onset [27]. From this study, no participant presented with an acute rubella infection.

The findings from our study further support the fact that rubella infection represents a public health burden. However, this study was subject to limitations including the small number of IgG negative and IgM positive cases, and no positive result after PCR. Due to limited resources, this cross-sectional survey was done only in two out of ten regions in Cameroon. Despite these limitations, our results highlight the need to investigate rubella during pregnancy, encourage the vaccination of young women of reproductive age presenting with no rubella immunity, and interpret IgM positive results carefully.

## Conclusion

The seroprevalence of rubella IgM antibodies was 5.0% in the pregnant participants. No participant presented with an acute/recent rubella infection, as confirmed with high avidity. Before taking any decision to interrupt any pregnancy because of a positive rubella IgM result, IgG avidity testing should be done. Our study reveals that susceptible pregnant women without immunity to rubella are present in the population and they are at risk of bearing children with CRS. Hence, rubella screening of women of child bearing age should be encouraged, those who lack immunity should be vaccinated, and rubella-based surveillance should be strengthened to reduce the burden of rubella and CRS in Cameroon.

## Supporting information

S1 Table Rubella seroprevalence Cameroon. (XLSX)
